# A cost analysis of postpartum home visit programming in Kenya: estimates to aid policymakers

**DOI:** 10.3389/frhs.2025.1644078

**Published:** 2025-11-13

**Authors:** Ednah Ojee, Joseph Odiyo, Judith Adhiambo, Eliza Mabele, Vincent Omondi, Emily R. Begnel, Bhavna H. Chohan, John Kinuthia, Soren Gantt, Dara A. Lehman, Jalemba Aluvaala, Fredrick Were, Vincent Were, Dalton Wamalwa, Jennifer Slyker

**Affiliations:** 1 Department of Pediatrics and Child Health, University of Nairobi, Nairobi, Kenya; 2 Adaptive Management and Research Consultancy, Nairobi, Kenya; 3 Research and Programs, Kenyatta National Hospital, Nairobi, Kenya; 4 Department of Global Health, University of Washington, Seattle, WA, United States; 5 Infectious Diseases & Immunology, CHU–Sainte-Justine Research Center, Department of Microbiology, Université de Montréal, Montreal, QC, Canada; 6 Human Biology Division, Fred Hutchinson Cancer Center, Seattle, WA, United States; 7 Policy, Research, and Programs, Kenya Pediatrics Research Consortium, Nairobi, Kenya; 8 Health Economics Research, African Population and Health Research Center, Nairobi, Kenya; 9 Department of Epidemiology, University of Washington, Seattle, WA, United States

**Keywords:** neonatal mortality, postnatal home visits, costs, staffing, universal health coverage, primary health care

## Abstract

**Introduction:**

The World Health Organization (WHO) and UNICEF recommend at least two postnatal home visits by a health provider within the first two weeks of life to improve newborn survival. Kenya's Universal Health Coverage (UHC) initiative includes a home-visit strategy to advance Sustainable Development Goal (SDG) 3.2, which targets reducing neonatal mortality to below 12 per 1,000 live births. We estimated the costs of starting a postnatal home-visit program in a level three health facility in Kenya, based on recommendations from Kenya's Ministry of Health policymakers.

**Methods:**

An ingredients-based costing method was used to determine the actual costs of home visits incurred during a research project conducted in 2019, and to estimate program costs from the government's perspective as the payer. Per-visit costs were calculated for three staffing approaches: Community Health Promoter (CHP), Registered Nurse (RN), and a Combined model where two providers (RN + CHP) visited each home together.

**Results:**

Staff salaries and transportation costs were the main drivers of recurrent program expenses. The CHP approach had the lowest total cost at $27,302 ($24.46 per visit), followed by the RN-only approach at $36.45 per visit, while the Combined model (RN+CHP) was the most expensive at $52.10 per visit. Discussions with policymakers noted that the RN+CHP approach was least feasible and scalable. They proposed an alternative “Hybrid” model in line with current programs being scaled up: weekly RN visits during the first month of life (neonatal period), and quarterly CHP visits thereafter.

**Discussion:**

This study presents a costing tool and generalizable formula that policymakers can use to estimate program costs based on different facility characteristics and staffing needs. The findings can support Kenya's efforts to scale up postnatal home-visit programs to improve maternal and newborn health outcomes within the UHC framework.

## 1 Introduction

Despite significant gains in reducing child mortality globally, neonatal deaths remain a considerable challenge—2.3 million newborns died in 2022, accounting for 47% of under-five deaths (1). A substantial proportion of these deaths occur within the first week of life, with 33% on the day of birth and 75% in the first seven days (1). In Kenya, the neonatal mortality rate (NMR) remains at 20.4 per 1,000 live births, well above the Sustainable Development Goal (SDG) of 12 (2, 28). Rural hospitals are particularly burdened, with neonates comprising up to 60% of inpatient deaths (3). These figures emphasize the need for practical, scalable, and context-appropriate strategies to reduce neonatal mortality.

Evidence supports the impact of postnatal home visits on improving newborn survival, particularly when integrated into community health platforms. The World Health Organization (WHO) and UNICEF recommend early postnatal home visits to enhance care-seeking and survival (4), and tools such as Essential Care for Every Baby (ECEB) offer teachable, community-adaptable frameworks (5). In Asia, community-based postnatal packages have reduced neonatal mortality by up to 50% (6–9). However, data on feasibility and cost-effectiveness in sub-Saharan Africa remain limited.

Where evaluated, costs of such interventions vary widely by country and delivery model. In Malawi, the community-based maternal and newborn care (CBMNC) package delivered by Health Surveillance Assistants (HSAs) costs under $2 per home visit, with national scale-up requiring 1.3% of public health expenditure (10). In Ghana, the Newhints trial found an annual cost of $203,998 and reported a higher than 70% probability of being cost-effective, influenced by baseline NMR and implementation efficiency (11). In contrast, higher costs were reported in South Africa, where the Goodstart III RCT found an estimated overall cost of $100 per mother, with each home visit requiring approximately 20% of this overall cost (12). A Bolivian analysis of a scaled maternal-newborn intervention estimated an average cost of $296 per mother-child pair visited (13).

Staffing models significantly affect both cost and outcomes. A cluster randomized controlled trial in Ghana paired Community Health Workers (CHWs) with Community Health Nurses (CHNs) to conduct three antenatal and postnatal home visits, resulting in improved care-seeking and skilled birth attendance (14). These findings suggest combining clinical and community skills may improve outcomes, but cost-effectiveness data remain limited.

Kenya has taken steps aligned with its Vision 2,030 goals to reduce neonatal deaths. These include increasing antenatal visits from four to eight, rolling out chlorhexidine for cord care, scaling up kangaroo mother care, and issuing national guidelines for newborn care (15–17). Postnatal services are offered through scheduled facility visits at 2, 6, 10, and 14 weeks, as well as at 6–24 months, linked to immunization schedules, wellness checks, HIV prevention, and family planning (18). However, many mothers miss the critical two-week postnatal visit, when most neonatal deaths occur (19, 20). Many newborns die at home without formal care or reach facilities too late for lifesaving intervention. To address this care gap, Kenya's Primary Health Care Strategic Framework (2019–2024) incorporated postnatal home visits as part of the Universal Health Coverage (UHC) platform (21). The framework relies on Community Health Promoters (CHPs), who receive a monthly stipend of approximately KES 5,000 ($38.91), and are tasked with conducting four quarterly home visits within a modified hub-and-spoke model. However, the cost implications and staffing strategies necessary to operationalize and scale this intervention remain unclear.

Kenya has effectively utilized home-visit programs as part of universal health coverage to improve health outcomes for various populations. These include supplementary immunization days, home-based voluntary counseling and testing for HIV, and follow-up for Non-Communicable Diseases (NCDs). Community Health Promoters (CHPs) are selected by the community, trained by nurses, and managed by a Community Health Assistant (CHA), who is a qualified nurse at level 2 (dispensary) and level 3 (health center). They receive a monthly stipend (ranging between KES 2,000 and 5,000), with half funded by the county government and the rest by the national government. This is implemented through the Kenya primary care networks (PCN), which utilizes a hub-and-spoke model, where the hub is a higher-level facility and the community health units (100 households assigned to a single CHP) at level 1 serve as spokes. The CHP service package in Kenya includes delivering essential health messages to households, registering and maintaining household records of births, vaccinations, and medical histories via the digital E-Community Health Information System (e-CHIS), and educating communities on health improvement and disease prevention. They identify infant clinical danger signs within households, refer individuals to health facilities when necessary, and diagnose, treat, and manage common uncomplicated infant and childhood illnesses such as pneumonia, diarrhea, and malaria. They also organize community activities, including health days, and specifically support postnatal mothers and their newborns by providing lactation support, cord management, and kangaroo care for small newborns.

While a recent multi-country analysis highlighted the importance of evaluating cost-effectiveness of community-based maternal and newborn care, Kenya was not included (22).There is insufficient data evaluating the costs of different approaches of delivering these postnatal home visits in Kenya. This lack of local economic data poses a barrier to evidence-informed policy implementation. This data is urgently needed to inform the modification of home visits and ensure they can be scaled to meet needs at a population level. To address this, our study draws on data from a longitudinal birth cohort to estimate the costs of delivering postnatal home visits from a payer perspective with the government as payer, exploring alternative staffing configurations and offering practical cost modeling tools for decision-makers.

The goal of this study was to fill a cost evidence gap for universal healthcare-aligned interventions in Kenya. Specifically, this study sought to determine the estimated cost of implementing postnatal home visits at the clinic level in Kenya, comparing different models, and to provide a simple, adjustable tool for policymakers to adjust to their varied program needs. We used real data to estimate the costs of implementing a postnatal home visit intervention at the clinic level in Kenya, and to compare the costs of different implementation models. Furthermore, we present a simple tool that allows policymakers to adjust program costs (capital and recurring) and program parameters (patient load, number of visits, provider cadre, etc.) to enable users to easily assess the impacts on implementation costs, thereby helping to guide program design. The results aim to inform the scale-up of postnatal home visits as part of Kenya's broader strategy to reduce neonatal mortality and strengthen primary health care under UHC.

## 2 Materials and methods

### 2.1 Study setting

We used budget records from the Linda Kizazi Study. In this prospective birth cohort study, pregnant women were enrolled during the third trimester of pregnancy and followed through two years postpartum between December 2018 and June 2023. Detailed study methods are described elsewhere (22). Briefly, we recruited women living with HIV and without HIV at Mathare North Health Centre, a Level 3 health facility located in a low-income, densely populated neighborhood of Nairobi, Kenya, and serving a population of nearly half a million. The Health Centre's Mother and Child health (MCH) clinic offers antenatal and postnatal services, including immunizations, well-child health and growth monitoring, contraceptive services, prevention of mother-to-child transmission of HIV services, comprehensive HIV care, and basic laboratory and pharmacy services. The MCH clinic workforce includes four nurses (2 in the antenatal clinic, one in the child wellness clinic, and one in the postnatal/family planning clinic), one clinician to see children, 10 nursing students, and four community health promoters (CHPs). The health worker to patient ratio varies based on workload, ranging from 1:40–1:60 based on patient volume.

### 2.2 Home visit procedures

All Linda Kizazi participants received clinic-based follow-up visits between week 6 and month 24 postpartum, aligned with the infant immunization schedule. Consenting mothers could additionally opt into weekly home visits for one year. Study staff included medical officers, clinical nurses, clinical officers, and CHPs. Study RN-CHP dyads conducted home visits; the RN-CHP dyad model was chosen for the research study home visits following consultation with the Community Advisory Board (CAB) who felt this model would be most appropriate for the community served (in contrast to a single woman or man visiting private homes on their own), and also afforded the visiting staff members more personal safety. Visits included a review of the mother-child health booklet, a brief physical examination, and a questionnaire assessing maternal and infant health, medication use, and nutrition. Mothers received lactation and nutrition counseling, education on infant danger signs, infant immunization, and, if living with HIV, HIV-specific counseling support, including prevention of mother-to-child transmission (PMTCT) of HIV. The RN-CHP team responded to any mother's concerns or questions, provided reminders about upcoming immunizations and study visits, and made referrals for additional medical care or support services as needed.

### 2.3 Policymaker engagement

A presentation of the home visit study was made to Policymakers to describe the research study home visits and discuss what information they felt would be helpful in a costing analysis *(qualitative manuscript in preparation)*. The Policymakers were included if they were directly involved in overseeing newborn, maternal, child and community health services or be involved in health budgetary allocation; they included leaders and administrators from Kenya's Ministry of Health working in the divisions of Maternal, Newborn, Child and Community Health selected from three administrative levels: National level (Ministry of Health programs), County level (Nairobi County health leadership) and facility level (Mathare North Health Centre administration). In alignment with policymakers' requests, estimates were generated for three different staffing approaches for a home visit program: Registered Nurses (RN), CHPs, and a Combined approach with an RN and CHP visiting together (RN + CHP). Preliminary discussions with Policymakers noted the RN + CHP approach was least feasible and scalable, and they proposed an alternative “Hybrid” model in alignment with current programs being scaled up: weekly RN visits in the first month of life (neonatal period), and quarterly CHP visits thereafter.The three models CHP-only, RN-only, and Combined models compared here were empirically selected by policy makers based on an iterative process from their reflection on our research model,the interviews and discussions held, their assessment of their currently existing health system/service delivery model since the inception of Universal Health Coverage in 2023 and a proposal to merge the best elements of both their existing model and our research model within limited resources resulting in their final proposal of the hybrid model (separate qualitative manuscript preparation further elaborates this).

### 2.4 Costing methods

*Key Assumptions:* Key assumptions are outlined in detail in Supplementary File 3. For Salaries, the research model used the actual research payment rates, which were higher than the government pay scale for personnel. The government model assumptions for personnel were as follows: For CHP, we used the prevailing government minimum wage rate as outlined in the Government Gazette notice for 2019, because currently CHP are not paid a salary but a stipend with no allowances for this category of minimum wage rate. For RN salaries, we used government salary scales for the year 2019 based on different job groups for basic salary and allowances. For time allocation, we interviewed nurses regarding time spent at work between the facility and community/home visits and used this total time spent on home visits as percentage of total time spent at work. We then allocated by percentage time spent by nurses who are assigned full time work in community/home visits; second category nurses assigned to both administrative duties and home visits and a third category of those who are fully assigned to facility work without home visits but redeployed or reassigned to home visits when the home visit nurses are on annual leave.

*Estimating Real Research Costs: We collected actual costs incurred from January 1 to December 31, 2019, covering a full financial year/time horizon without interruptions or program changes.* Cost data was extracted from the study and accounting records and managed using Excel v2016 (Microsoft Corp). Spreadsheets were programmed to categorize cost components and apply formulas to calculate total costs, per visit, and overall analysis. We calculated the cost of research-provided postnatal home visits using an ingredients-based costing approach from the payer perspective of the research study. The cost per visit was determined by dividing the total cost by the number of visits. The costs analyzed reflect the actual expenses incurred in delivering these home visits.

Resources were categorized as capital (buildings, vehicles, equipment, one-off training) or recurrent (personnel, supplies, transport, maintenance). Shared resources were allocated proportionally based on the ratio of home visits to total visits. The time horizon was 12 months (FY 2019), with data collected from financial records and staff interviews. For this analysis, the 2019 US Dollar exchange rate against the Kenya Shilling (KES) used was 101.99 KES = 1 US Dollar during the selected time horizon of January to December 2019. The nominal exchange rate was used throughout the calculation, except for capital costs for both research and government, where adjusted exchange rates applied (adjustment for inflation included).

The allocation of Costs for home vs. clinic-related costs was derived from the relative number of visits. The resource utilization by a home or clinic visit is assumed to be proportional to the number of home and clinic visits conducted. Home and clinic visit-specific costs were allocated directly at 100% to the Linda Kizazi home and clinic visit. Shared costs between home and clinic visit were assigned to the home visit at 82.7%, based on the number of home visits in 2019 (1,116) as a proportion of the total visits for Linda Kizazi Project in 2019 (1,350), and at 17.3% to the Clinic visit, based on the number of clinic visits in 2019 (234) as a proportion of all visits for Linda Kizazi Project in 2019 (1,350). The research cost per home visit accounts for 82.7% of the total project cost allocated to home visits in 2019. This amount is then divided by the total number of home visits (1,116) to determine the cost of a single Linda Kizazi Home visit.

*Estimated Government Payer-Perspective:* As requested by the policymakers, we estimated costs for programs staffed by RN, CHP, or a combination of RN and CHP. Mathare North Health Centre was assumed to be the administrative base for all analyses of home visits. Capital costs were held constant when estimating the cost from the government's perspective for different staffing approaches. At the same time, adjustments were made for staff salaries and additional transport costs for nurses, following Ministry of Health (MOH) guidelines. These estimates were obtained from published Kenyan guidelines for human resources for health workers and WHO guidance. We developed a calculator in Excel to enable Policymakers to easily adjust capital and recurrent costs to estimate 1st year program costs for different-sized programs.

### 2.5 Sensitivity analysis

We performed a one-way sensitivity analysis on the cost outcomes to evaluate how changes in key cost drivers—components most likely to vary if the intervention is scaled up in different settings—affect government-estimated implementation costs. For this analysis, we varied RN and CHP salaries in their respective models; nurse salaries were adjusted by ±4% based on the Kenya Salary Remuneration Commission (SRC) 2019 salary scale. The CHP stipend was varied between (2,000–5,000), with the lower value representing the pre-2024 county payment rate and the higher value reflecting the current stipend shared equally by the county and national governments. Additionally, we conducted a sensitivity analysis assuming the CHP stipend is replaced by the current government minimum wage rate of KES. 16,113.75 (Regulated Wage Order, 2024). We estimated the highest and lowest cost scenarios for salary adjustments within each cadre. The least costly scenario for the RN model involved reducing nurse salaries by 4%, while the costliest involved increasing salaries by 4%. For the CHP model, the least costly scenario utilized the pre-2024 stipend, while the most expensive scenario employed the adjusted stipend after 2024, along with the current government minimum wage rate.

## 3 Results

### 3.1 Real costs of research study

#### 3.1.1 Participant characteristics

**Figure 1:**
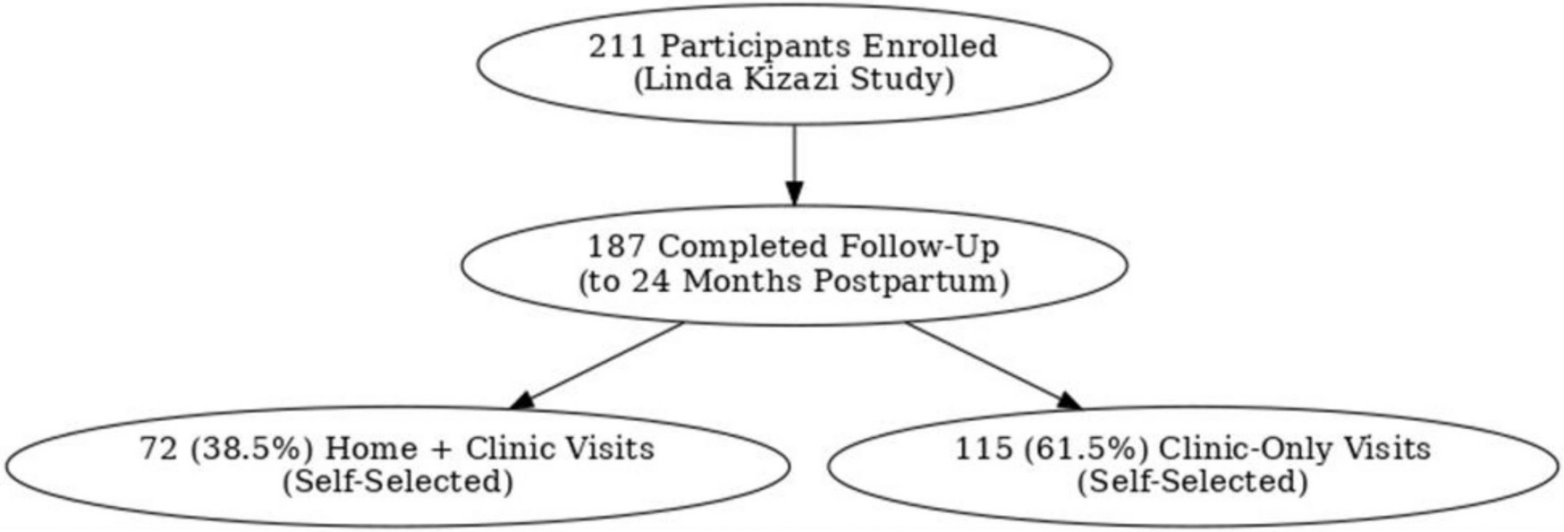
Flow diagram showing participant self-selection of visits in Linda Kizazi study (home visits with clinic visits and clinic visits only).

Two hundred eleven (211) mother-infant pairs were enrolled in the parent cohort (Linda Kizazi); 187 (89%) had postpartum follow-up data (Figure 1 illustrates this as a flow diagram). Specifically, 72 (39%) received weekly home visits, while 115 (61%) received only clinic visits. Supplementary File 1 under the Background Information tab has a table summarizing the characteristics of the participants outlined, comparing the home visits to the clinic-only group. The mothers self-selected whether to receive the additional weekly home visits or clinic visits only. Among the 187 participants with complete follow-up data, 72 who received home visits were slightly younger, with a median age of 26 (24–30) years, compared to 115 in the clinic-only group, with a median age of 29 (26–33) years. Mothers who received home visits who were married/in steady relationship were 68 (94%) compared to clinic group 105 (91%); fewer mothers receiving home visits 24 (33%) were employed compared to the clinic group at 57 (49%) and fewer were living with HIV 28 (39%) compared to clinic group 58 (50%)Among mothers in the home visits group, fewer than 42 (58.3%) had a secondary education or higher compared to the clinic group, 55 (47.8%).In terms of crowded living circumstances, the home visits group was 48(67%), similar to the clinic group, 74 (69%).

#### 3.1.2 Research visits

Supplementary File 1-(under Background information tab). Overall, during the five-year study period, a total of 3,575 visits were recorded, comprising 2,327 (65.1%) home visits and 1,248 (34.9%) clinic visits. In 2019 (the year of costing) specifically, there were 1,350 total visits, comprising 1,116 (82.7%) home visits and 234 (17.3%) clinic visits provided (Supplementary File 1 under Background information tab).

### 3.2 Research study costs

The total cost of the study, which involved 1,116 postnatal home visits in 2019, was 11,476,394.92 KES (approximately USD 108,445.48), resulting in a cost per visit of 10,283.50 KES ($97.20). Table 1 lists the components used for estimating capital and recurrent costs. Supplementary File 1 (Under Costing Method and Allocation Tab) provides costing data and formulas. When these costs are combined, they yield the total research cost to deliver the 1,116 visits in 2019. Capital resources totaled 466,246.35 KES (approximately USD 4,571.49), comprising building space, vehicles, equipment, digital electronic tablet, and initial training. Recurrent resources totaled 11,010,149 KES (USD 107,953), with personnel being the most significant component at 8,293,344 KES (USD 81,315), followed by transport at 1,085,700 KES ($10,645).

**Table 1:** Total resources and cost per home visit.

(a) Cost of Capital and recurrent Resources in KES and USD		
Capital Resources	KES	USD
Building (container office)	75,478.15	740.5
Program Vehicles	21,206.06	207.92
Other Capital Equipment	273,027.37	2,677
Tablets for Home Visits	39,244.1	385
Initial Training/Mobilization	49,262.01	483.01
Total Capital Cost	458,217.69	4,492.77

Recurrent Costs and Totals (KES and USD)		
Recurrent Resources	KES	USD
Personnel	92,05,872	90,262.5
Transport	11,51,787	11,293.14
Utilities and Overheads	435,965.03	4,274.59
Supplies	862,765.49	8,451.31
Recurrent Trainings	–	–
Building Rental	294,742	2,889.92
Total Recurrent Cost	11,95,1132.32	117,179.45
Total (Capital + Recurrent) Costs	12,40,9350.01	121,672.22
Study cost per research study visit	11,119	109.03

(b) Cost of Capital and Recurrent Resources in KES only	
Capital Resources	KES
Building (container office)	75,478.15
Program Vehicles	21,206.06
Other Capital Equipment	273,027.37
Tablets for Home Visits	39,244.1
Initial Training/Mobilization	49,262.01
Total Capital Cost	458,217.69
Recurrent Resources	
Personnel	92,05,872.0
Transport	11,51,787.0
Utilities and Overheads	435,965.03
Supplies	862,765.49
Recurrent Trainings	–
Building Rental	294,742.0
Total Recurrent Cost	11,95,1132.32
Total (Capital + Recurrent) Costs	12,40,9350.01
Study cost per research study visit	11,119

(c) Cost of Capital and Recurrent resources in USD only)	
Capital Resources	USD
Building (container office)	740.5
Program Vehicles	207.92
Other Capital Equipment	2,677.0
Tablets for Home Visits	385.0
Initial Training/Mobilization	483.01
Total Capital Cost	4,492.77
Recurrent Resources	
Personnel	90,262.5
Transport	11,293.14
Utilities and Overheads	4,274.59
Supplies	8,451.31
Recurrent Trainings	–
Building Rental	2,889.92
Total Recurrent Cost	1,17,179.45
Total (Capital + Recurrent) Costs	1,21,672.22
Study cost per research study visit	109.03

### 3.3 Estimated government capital and recurrent costs for 3 staffing approaches

Using our research costs to create a list of program cost “ingredients,” we next estimated the cost of establishing and operating a program from the government payer perspective to provide the same number of research visits (Table 2). Supplementary File 2 includes the Excel sheet with data analysis based on the cost of ingredients and financial expenses, modeled after government pay scales and expenditures, to determine recurrent costs. The capital costs for the government model were maintained as constant, assuming our estimated capital start-up costs were low, due to the utilization of existing government structures, such as buildings and vehicles, with minimal need for additional expenditure. The primary difference from the research model was the recurrent costs; staff salaries had greater financial resources and spending in the research setting than the government model's lower resources and expenditures. The combined approach (RN + CHP) was the costliest, costing +$27.64 more per visit than the CHP-only model, followed by the RN-only model, which cost +$11.99 more per visit than the CHP-only model.

**Table 2a:** Payer as government: capital costs for three home visit staffing approaches.

Capital Resources	KES	USD
Capital Building	75,478	740
Capital Program Vehicles	21,206	208
Other Capital Equipment	2,73,027	2,677
Total Capital Cost	3,69,712	3,625

The government scale-up assumes use of existing administrative infrastructure, avoiding the additional capital investments required in the research setting. Costs such as digital tablets and initial training—incurred in the research model—were excluded, based on the assumption that the government already provides tablets through the e-Community Health Information System (launched in 2023 with UHC) and includes training within existing budgets.

**Table 2b:** Recurrent costs by staffing approach with the payer as the government.

Component	RN Only (KES/USD)*	CHP Only (KES/USD)*	Combined (KES/USD)*
Personnel	3,726,906 (36,542)	1,824,120 (17,885)	2,943,726 (18,037)
Utilities & Communication	444,319 (4,356)	444,319 (4,356)	444,319 (4,356)
Overheads (CHP kit)***	750,000 (7,354)	750,000 (7,354)	750,000 (7,354)
Transport	830,322 (8,141)	N/A	830,322 (8,141)
Total Recurrent Resources**	3,778,763 (37,050)	2,414,812 (23,667)	5,563,151 (54,546)

The difference in cost with personnel and transport (approaches with RN require transport allowance as they do not live within the communities they serve);while CHP approach does not require transport allowance as it is assumed that they live within their communities and walk to their assigned households as with the current UHC model in Kenya.

* Nominal exchange rates used for 2019 as KES 101.99 to 1 USD throughout the calculation except for **where adjusted exchange rate applied.

* NA-Transport costs do not apply to community health promoters (assumption that they live within the communities they are assigned to and walk to the homes, not incurring transport costs).

* Estimates for providing 1,116 home visits.

* RN-Registered government nurse only.

* CHP Community Health Provider Only.

* Combined- When both the Government Registered Nurse and the Community health promoter conduct visits together/ concurrently.

**** CHP kit contents: Complete details of contents outlined in Supplemental file two under supplies, including Essential Equipment, Tools for Household Screening, data collection, First Aid, Communication and support, smartphones with e-community health information system.

** Recurrent cost per home visit (instead of the final total per visit) is applied in Equation 1 for cost per visit to avoid double calculation with capital cost, which is initially maintained as constant in the equation.

**Table 2c:** Total cost and cost per visit by staffing approach.

Component	RN Only	CHP Only (Ref.)	Combined (RN + CHP)
Total Capital Resources KES (USD)	369,712 (3,625)	369,712 (3,625)	369,712 (3,625)
Total Recurrent Resources KES (USD)	3,778,763 (37,050)	2,414,812/23,667	5,563,151 (54,546)
Total Cost KES (USD)	4,148,474 (40,675)	2,784,524 (27,302)	5,932,863 (58,178)
Cost per Home Visit KES (USD) with [Confidence interval]	3,717.3 (36.45)	2,495.1 (24.46)	5,932.9 (52.12)
	(28.69–43.96)	(19.7–29.6)	(41.15–63.04)
Recurrent Cost per Home Visit KES (USD)	3,386 (33.20)	2,163.81 (21.22)	4,985 (48.88)
Cost Difference (USD)	+11.99	Ref.	+27.64

Supplementary File 3 (containing Supplementary Tables S4a,b,c) has been included, which summarizes the cost categories, indicating how shared costs were allocated and the assumptions made. Supplementary File 3, Supplementary Table S5, summarizes the costing methodology, included in the Excel calculator Supplementary File 1, used to calculate the research cost of postnatal home visits under the “Cost Method & Allocation” tab.

Cost allocation between home and clinic visits was based on the relative number of visits. Resource use was assumed to be proportional to the number of visits conducted. Costs specific to home or clinic visits were fully allocated (100%) to their respective categories. Shared costs were divided based on visit volume: 82.7% to home visits (1,116 of 1,350 total visits in 2019) and 17.3% to clinic visits (234 of 1,350). The research cost per home visit was calculated by allocating 82.7% of the total project costs to home visits, then dividing by the number of home visits (1,116) to determine the cost per home visit.

**Figure 2:**
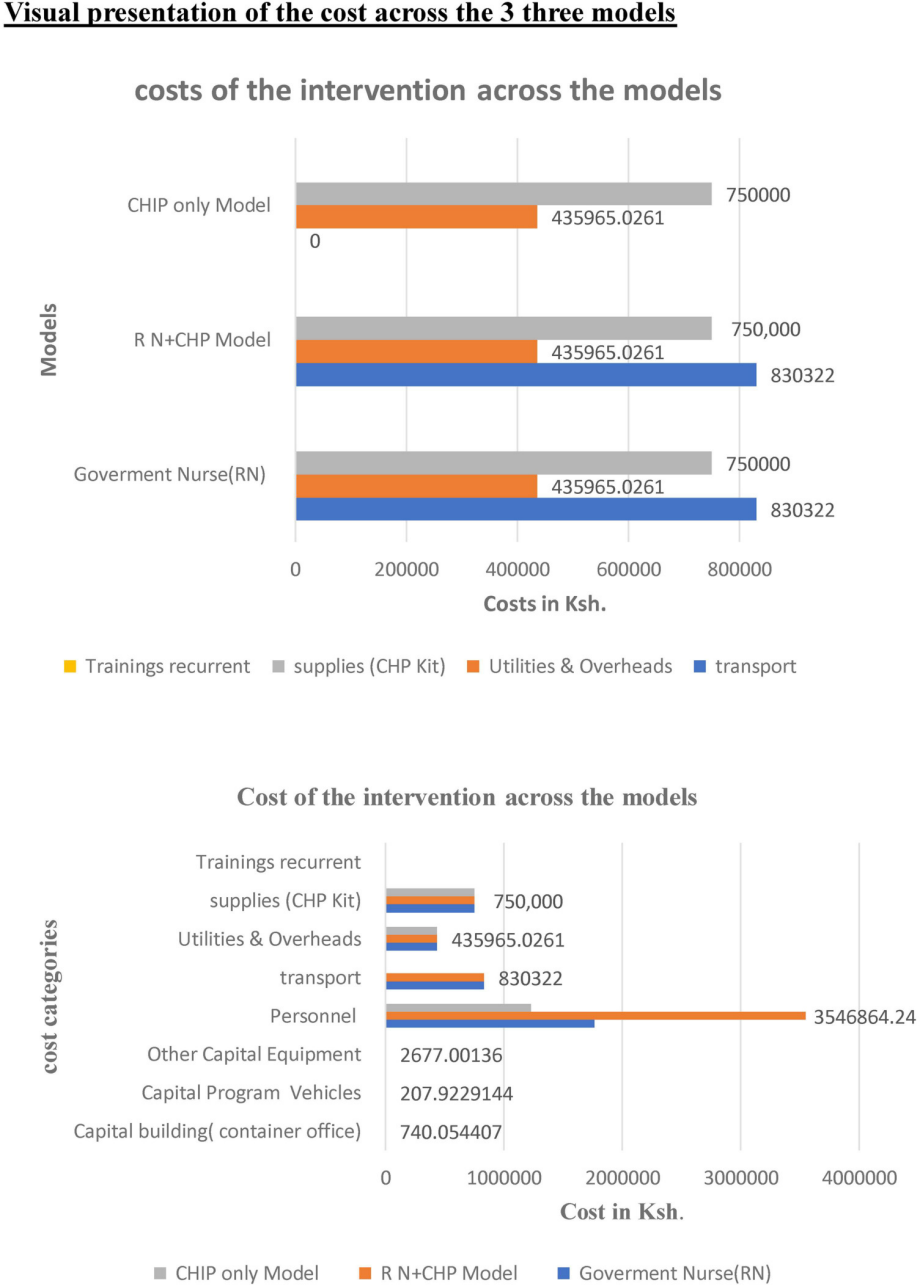
Comparison of cost categories across three models RN only CHP only and combined, illustrating the cost drivers of the post-natal home visits intervention.

*Key cost drivers:* The key cost drivers for all models were salaries and transportation (Figures 2a,b, respectively, illustrate a comparison of cost categories and cost drivers across the three models using bar graphs). Personnel costs accounted for a larger proportion of the RN + CHP and RN-only program costs compared to the CHP-only program.

### 3.4 A general model for estimating program costs for establishing, maintaining, or scaling up a program

Policymakers recognized that the combined approach, where nurses and CHPs conduct home visits in pairs, would not be feasible or scalable due to limited funding for employing health workers, staff shortages, and associated costs. They requested that we provide cost estimates for a modified program in which nurses carry out the first, highest-risk visits weekly during the neonatal period. CHPs would conduct the remaining visits as envisioned in the rollout of UHC (a hybrid approach). This differs from the current provision, where CHPs conduct home visits to families covering a diverse population with a broad scope, including quarterly visits for postnatal mothers and babies, geriatrics, and family members with chronic illnesses. Additionally, they requested that the estimates be flexible to incorporate additional pro re nata (PRN) RN visits when CHPs encounter complex cases and require a second opinion from a healthcare professional.

**Figure 3:**
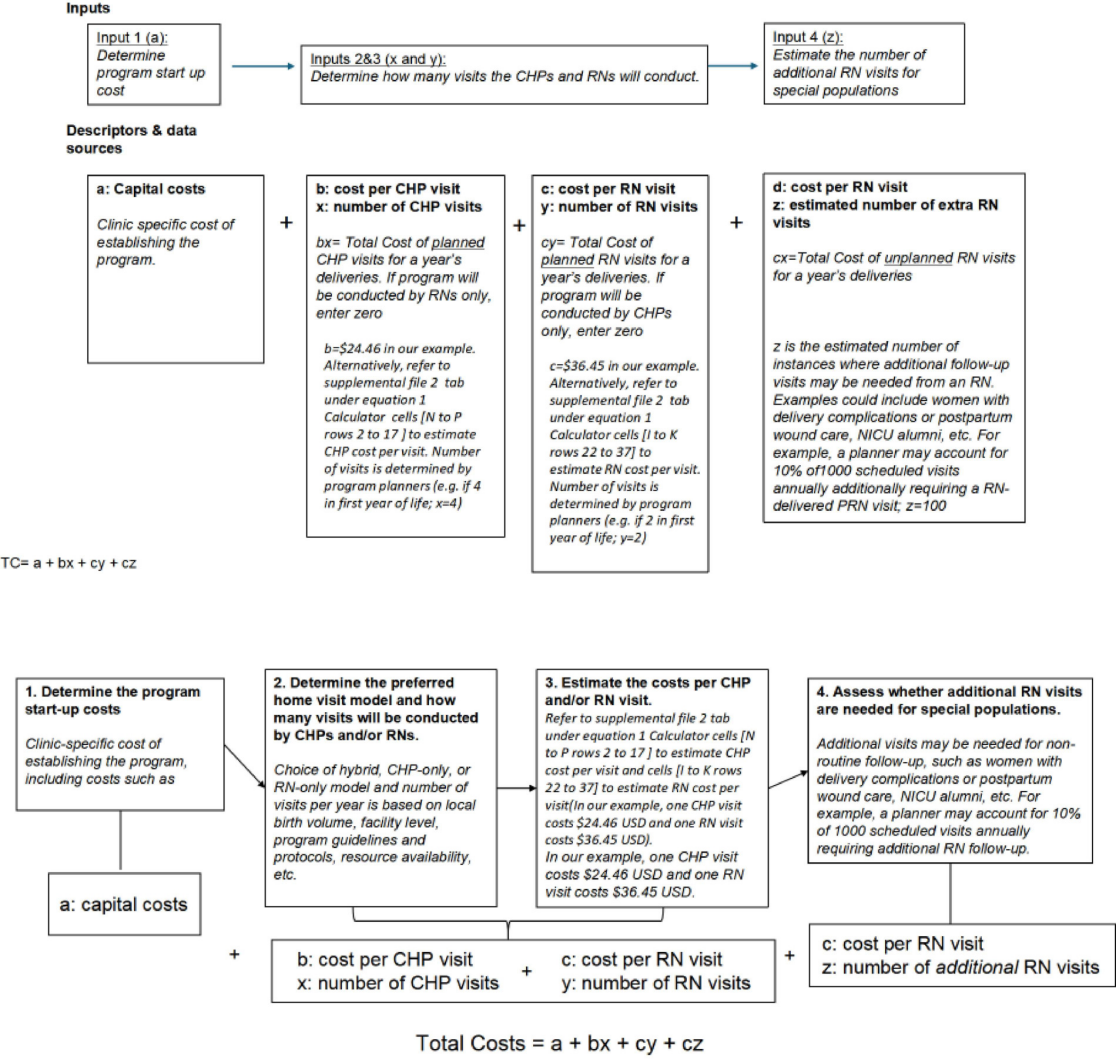
Flow diagram to guide the use of cost calculators.

We present a simple equation (Equation 1) with accompanying flow diagram (Figure 3) to enable policymakers to estimate their costs for launching and expanding a home visit program by adjusting the cost per visit based on specific contexts and staffing choices. Capital costs can vary depending on the characteristics of individual clinics and actual costs from facility budgets. For example, our estimated capital start-up costs in Table 2 were low, assuming existing structures, staff, and vehicles were used. Still, these could be adjusted to account for procurement as necessary. To utilize the calculator for starting a program in a different setting, one can enter customized cost data into the Excel sheet tabs for personnel, supplies, and transport, then transfer the total recurrent costs to the final equation and add them to capital costs. The costs of home visits can be modified based on actual transportation expenses and distances for different urban, semi-urban, and rural areas with varying catchment zones. The number of visits can be estimated by the average annual number of deliveries, adjusted for the number of home visits per client the clinic wishes to implement. Since clinics may want to deploy higher-level healthcare staff for complex medical cases or more vulnerable families, these estimates can also be easily adjusted based on the predicted proportion of visits a clinic might want to carry out.

Equation 1: General Costing formula/model to estimate costs for the first year of the home visit program.

TC=a+bx+cy+cz (1)

Where:

**TC,** Total cost; **a**, Fixed capital cost; **b**, Cost per CHP visits; **c**, Cost per RN visit; **x**, Number of CHP-only visits; **y**, Number of RN only visits; **z**, Number of additional RN only visits.

**Notes. PRN = pro re nata, second visits conducted by an RN for complex cases following an initial CHP visit. Startup and recurrent costs per visit can be generated de novo for new sites from Supplementary File 2. From this equation, policymakers can estimate any program iteration based on local needs.

***The recurrent cost per visit (Supplementary Table S2 and Supplementary File 2) is used in this equation because capital costs are maintained as constant at the beginning, thus avoiding the double inclusion of capital costs.

### 3.5 Worked example of costing the hybrid postnatal home visit program to inform a budget impact analysis

In this example, a county aims to determine the costs of launching a new program that serves 100 women per month (1,200 clients per year) at a level 3 facility, estimating eight visits per client (four visits conducted in the first month of life by RNs (4,800 visits), followed by four quarterly visits by CHPs (4,800 visits). Based on the patient population, the county estimates that 3% of CHP visits will require a second visit by an RN due to complex medical or social situations identified by the CHP during the first visit. For simplicity, we will use our estimates from Table 3, assuming low start-up costs due to existing equipment and infrastructure. The total cost for the first 1,200 patients to receive their 14 visits would be $300,294.

**Table 3:** Comparison of the scope of interventions on postnatal home visitstable.

Country/Study/Year/Journal	Study Design/Population	Intervention Scope	Training/Supervision	Cost per Visit/Annual Cost
Ethiopia (COMBINE)	Cluster RCT; Health Extension Workers (govt paid)	Preventive & curative care with ICCM training	1.5 days ICCM within 6-day program; monthly supervision	CHW $891/yr, vCHW $74/yr; Cost per visit: $22.70
2017 Oxford Health Policy and Planning				
Malawi (CBMNC)	Government tracking + before/after survey; Health Surveillance Assistants (govt paid)	Mobilization & preventive care CBMNC training	10–12 weeks basic + 21-day facility + 10-day CBMNC training	$1,445/yr plus $205 training
2017 Oxford Health Policy and Planning				
South Africa (Goodstart II) 2017 Oxford Health Policy and Planning	Cluster RCT; Goodstart CHWs (study recruited paid)	Preventive care with 10-day training	10-day training with local recruitment	$35,714/yr plus training costs
Tanzania (INSIST) 2017 Oxford Health Policy and Planning	Cluster RCT; Mtunze CHWs (volunteers)	Mobilization with 5-day training	5-day training by govt health staff; 4 CHWs trained, 2 selected	Range $12.60–$19.50 per visit
Uganda (UNEST) 2017 Oxford Health Policy and Planning	Cluster RCT; Volunteer health teams (community recruited)	Preventive package with 5-day training	5-day training; supervision by District Health Teams	$34 intervention arm; $27 control arm per visit
Ghana (Newhints) 2017 Oxford Health Policy and Planning	Cluster RCT; Research-based surveillance volunteers	Preventive with clinical assessment at home	8 days total (3 + 4 + 1 refresher); monthly supervision + group meetings	$3.49/month training incentive
Bolivia 2017 Oxford Health Policy and Planning	Government tracking + before/after survey; Previously recruited govt workers	Mobilization (no additional training)	No additional training; 1 supervisor per 35 CHWs	Minimal additional costs (existing workers)
**Kenya (Linda Kizazi)—CHP only	*Prospective cohort study; Community Health Promoters*	*Preventive with referral and linkage to a health facility*	*Initial training on Home visits at the start of research*	*$26.51 per visit*
			*CHP training and supervision protocols*	
***Kenya (Linda Kizazi)—RN only	*Prospective Cohort Study: Registered Nurses*	*Preventive with referral and linkage to a health facility*	*Initial training on Home visits prior to of research*	*$36.45 per visit*
			*Professional nursing qualification and ongoing supervision*	
***Kenya (Linda Kizazi)—Combined (RN *+* *CHP)*	*Prospective Cohort study; Combined RN* *+* *CHP model*	*Preventive with referral and linkage to health facility*	*Initial Training on Home visits prior to start Research Combined RN and CHP training*	*$52.12 per visit*

**Findings form this current study which are yet to be published to distinguish form the previous studies which are published.

*** Kenya Unpublished-Findings from this current study.

In our example, applying the formula.

TC=a+24.46x+36.45y+cz

The coefficients represent the unit cost per visit for each staffing model,

b = 24.46 (CHP).

c = 36.45 (RN).

Total program costs = $2,677 + (4,800 × $36.45) + (4,800 × $24.46) + (.03 × 4,800 × $36.45) = $300,294.

Further examples of cost calculation with varied iterations for staff conducting visits are provided in Supplementary File 2 (Equation 1 calculator tab). For example, the cost when all eight visits are undertaken solely by CHPs is much lower than that of a hybrid program ($272,485). However, such a program may require more PRN visits from secondary nurses during the neonatal period; therefore, the savings from CHP-only visits might be offset by the increased need for PRN RN visits. In a scenario where an RN provides all eight trips, the cost is highest ($352,957), but this approach reduces the necessity for PRN visits and may lead to better outcomes. While the RN-only cost may be prohibitive for an entire program, this strategy might suit specific high-risk populations. For example, a program could primarily utilize CHPs but concentrate RN staffing on select case populations.

### 3.6 Sensitivity analysis

The sensitivity analysis results showed that increasing the salary of nurses working in the RN only model by 4%, increased the cost per home visit intervention by 2% to $37.07 from $36.45. In the CHP only model changing their stipend from an estimated range between $19.60 to $49.02 (KES 2,000–5,000) increased the cost per home visit intervention by 6% to $26.05 from $24.45 while replacing their current stipend with the government's current minimum wage rate increased the cost per home visit by 56% to $38.05 from $24.45. In the highest and lowest cost scenarios, for the RN-only model, the unit cost varied between ($35.83–$37.07) and for the CHP-only model, the unit cost of the home visit varied between ($22.88–$26.05) and ($22.88–$38.05) when the stipend and minimum wage are used, respectively. This applies to both the combined model for CHP and RN, as well as the hybrid model.

## 4 Discussion

Home visit programs are being considered in Kenya and other countries to reduce neonatal and infant mortality. While this is an evidence-based approach to improving infant survival, few costing studies have been conducted to date. Countries could implement various staffing strategies and visit schedules to expand such programs. In this study, we used real data from a birth cohort to estimate the cost of a government-run postnatal home visit program. We also present a simple cost calculator with adjustable inputs to help policymakers in program planning. Importantly, the tools and estimates provided in our study offer flexibility for governments to adjust and estimate costs based on local staffing, transportation, and operational realities.

Our study's cost calculator complements standardized tools like WHO CHOICE by offering micro-level, program-specific cost analysis tailored for researchers and institutions. While WHO CHOICE provides macro-level estimates to inform policymakers and health system planners in setting priorities and allocating resources, our tool supports financial planning and budgeting at the program/project level, making both tools valuable and mutually reinforcing in health intervention costing. Policymakers can utilize these standardized costing tools to tailor program budgets to their specific needs and resources, ensuring efficiency and sustainability in scaling up postnatal home visit programs. A Supplementary File 4 is provided to outline further how our costing tool integrates with other standardized tools, in comparison to the WHO CHOICE. This costing of the home visit intervention was conducted using a standardized framework adapted from WHO-CHOICE (Karin Stenberg et al. 2021), and costs were estimated from the perspective of the government as the health system funder. The analysis includes patient-level service delivery costs (example contents of the CHP kit including thermometers, oral medicine for treating uncomplicated illnesses such as malaria, pneumonia, and diarrhea) and broader programmatic costs such as personnel, materials and supplies, transport, maintenance, and utilities. Health system-level costs were limited to capital inputs only—specifically, the cost of the service delivery container, which served as an administrative office within the health center. Costs incurred by patients (e.g., transport fees) were not included, as services were provided through community visits.

Although our cost calculator was developed at a micro (programmatic) level and was not designed for system-level cost-effectiveness analysis, it conceptually aligns with the input-based structure used in WHO-CHOICE. Specifically, inputs were organized into three standardized categories: labor, capital, and recurrent expenditures. Labor costs captured salaries and benefits for frontline health workers such as registered nurses (RNs) and community health providers (CHPs). Capital costs included long-term investments such as training, equipment, vehicles, and infrastructure, annualized over their expected useful life. Recurrent expenditures comprised items like consumables, utilities, maintenance, transport, and administrative overheads. Supplementary Table S6 outlines the mapping of our cost categories to the WHO CHOICE tool as a standard tool. The Consolidated Health Economic Evaluation Reporting Standards (CHEERS) checklist has been included as Supplementary File 5.

Several programs across Africa have implemented postnatal home visit initiatives, with reported costs varying widely based on program design, staffing, and local context. Evidence indicates that the costs of these interventions differ significantly by country and delivery model. A multi-country economic analysis of seven countries (Summarized in Table 3), which included three East African countries but excluded Kenya, applied a standardized costing methodology from the COIN (“Cost of Integrated Newborn”) Care Tool, developed by the South African Medical Research Council and Saving Newborn Lives Network, for evaluating community-based maternal and newborn (CBMNC) care programs. This multi-country analysis found that the cost of home visits ranged as follows: Tanzania ($12.60–$19.50), Uganda ($34 for the intervention arm; $27 for the control arm), and Ethiopia ($22.70). They noted that community-based approaches consistently had lower costs, with labor staffing choices significantly impacting overall expenses, while program setup and training contributed substantially to the overall cost. Similarly, our study revealed lower operational costs in the CHP approach ($24.46) but higher costs for RN ($36.45), justified in complex cases requiring higher qualifications. Our increased overall costs can be attributed to transport facilitation, staff salaries, and comprehensive service packages (23–25).

Our cost analysis showed that the CHP-only staffing approach was the least costly at $24.46 per visit. This efficiency comes from the low stipends given to CHPs, the absence of benefits, and lower overhead and travel costs, which help keep community access open. This approach aligns with evidence suggesting that community health promoter/worker programs are a very cost-effective way to deliver components of primary healthcare in resource-limited settings (26). Additionally, the CHP method has strong potential for scaling up. In our study, an RN-only home visit program cost $11.99 more per visit than a CHP-only program. This higher cost is mainly due to higher salaries and benefits for RNs and the need for transportation assistance from health facilities to households. While these staffing options are ideal in terms of expertise, they are often unrealistic in Kenya and similar places because of healthcare worker shortages and limited budgets (27–30). Although the RN + CHP combined model provides better provider safety and high community support, policymakers said it was not practical or scalable because of the high cost.

The range of reported costs for CHP-staffed programs is lower, whereas for RN-only programs, it is higher. Our estimated costs for RN and RN + CHP-staffed programs were greater than those reported for CHP-staffed programs. This was primarily due to the need for transport facilitation for nurses and the higher salaries for RNs in these models.

Unlike other studies where transportation was not a significant cost driver (because CHPs tend to work in the communities where they reside), employing nurses who travel from a centralized clinic requires substantial transport support, thus increasing the overall program costs. Our costs exceeded those of other CHP-only programs, likely because our analysis focused on direct operational costs. In contrast, other studies may have included broader supervisory and management expenses. Policymakers potential highlighted from our research model need to integrate alternative cost saving approaches to address the high cost of transport facilitation for example telehealth form our research setting which was applied during COVID 19 when in person visits could not continue in line with prevention guidelines (A separate qualitative manuscript is in preparation highlighting policy makers perspectives on scaling up postnatal visits).

This report provides policymakers with a reference framework and tool to assist in budget projections for scaling up postnatal home visits, with modifications within the Ministry of Health's (MOH's) existing model, aligned with global recommendations. During formative work, policymakers noted that the ideal program would be to deploy nurses for the home visit programs. Still, this staffing approach is infeasible in Kenya and many other countries due to high costs and healthcare worker shortages (27–30). They proposed a program model that utilized RNs for high-risk periods or households and was flexible to the need for PRN visits following CHP encounters. Our estimates demonstrate the benefits of this approach, which focuses on higher-level care where it is most needed and utilizes CHP for the lowest-risk patients. One could use our tool to estimate a program that deploys CHPs to low-risk households and RNs to higher-risk households, thereby achieving a tradeoff between cost and clinical expertise. Such high-risk populations could include complicated deliveries, alumni of neonatal intensive care units, neonates discharged with congenital medical conditions, neonates born preterm, and families with very high socioeconomic vulnerability.

When registered nurses (RNs) conduct home visits during the early neonatal period and identify newborns with complications requiring hospital care, early referral enables timely intervention. This can result in shorter hospital stays and reduced healthcare costs by preventing complications associated with delayed treatment. The trade-off involves the additional cost of deploying RNs for early neonatal home visits vs. the potential savings from reduced hospital stay duration and severity of illness.

The hybrid model, as proposed by policy makers, allocates different types of health workers based on household or newborn risk level, and offers a promising balance between clinical effectiveness and resource efficiency. While direct evidence quantifying its impact on neonatal mortality is currently limited, plausible outcomes can be inferred from patterns observed in targeted interventions. By assigning more specialized providers, such as registered nurses, to newborns or families with identifiable risk factors—such as medical complications at birth, preterm delivery, or socioeconomic vulnerability—the model is positioned to improve early detection and management of severe complicated conditions. This may lead to earlier hospital referrals, reducing the severity of illness and shortening hospital stays, which in turn could lower the overall cost burden of inpatient care.

On the other hand, deploying community health providers to households with lower risk maintains broad coverage without overstretching limited skilled clinical resources. This stratified approach helps maintain feasibility at scale by conserving high-cost human resources while still enabling system responsiveness to preventable mortality. The trade-off lies in upfront investment for targeted risk screening and workforce coordination, but these costs may be offset by downstream savings and improved health outcomes if implemented effectively. Future modeling and real-world testing would be valuable to quantify mortality reduction and cost-efficiency gains under this approach.

Similar to findings from Malawi, our analysis highlights the CHP approach as the most cost-effective for scaling up routine postnatal home visits (10).In contrast to a study in Ghana, where program set up and implementation costs were a key driver, startup costs were lower in our study. In contrast to Ghana, our study found that nurses' salaries and transport facilitation were key cost drivers in the RN approach, with savings in the CHP approach requiring lower wages/benefits and minimal transport cost (11). A South African study had a lower cost than our RN approach, but approximately the same cost as our CHP approach. The affordability of both CHP approaches in different countries reinforces the scalability of interventions where CHPs serve as the primary providers at the community level (12).In contrast, a Bolivian study estimated a much higher visit cost than ours at $296 per mother-child pair (our cost being lower by eight and twelve times, respectively, for RN and CHP approaches). Bolivia's highest cost incurred was salaries, as with ours, but with an additional cost for supervision. This Bolivia study raised critical questions about the efficiency of high-cost interventions towards reducing neonatal mortality and improving care-seeking behavior. It offered insights into seeking alternative, affordable, and feasible staffing approaches for scale-up (13).

A multi-country analysis from these nations supports the notion that increased program delivery costs can lead to improved outcomes through higher-level cadres. In five countries, they further identified that key cost drivers included the number of Community Health Workers (CHWs) and their availability. Fixed costs per CHW accounted for over 96% of the total expenses, covering equipment, training, salaries, supervision, and management (22). Our findings align with this multi-country analysis concerning the significant role of personnel salaries as a cost driver, with RN salaries being the highest. Yet, the lowest in our CHP-only approach, and the combined model proves to be the most expensive, making it infeasible and unscalable. Consequently, policymakers recommend an alternative hybrid approach.

Policy makers emphasized the need to integrate this scale-up within the existing community health service delivery model that has been rolled out through Universal Health Coverage (UHC). This approach will be highly cost-effective and will reach families of low socio-economic status, including the most difficult-to-access women and their babies (qualitative manuscript in preparation). However, they underscored the importance of considering additional factors in the scale-up process. They recommended broadening the planning framework for sustainability by taking into account multiple factors, including cadre, supervision, content of the package, supplies, commodities, linkage to facility care, and financial incentives for CHPs. Policy makers also highlighted the risk of overburdening CHPs through task-shifting and stressed the need for incentives to sustain performance. To address this, they proposed a hybrid model where nurses manage the more critical neonatal period, while CHPs conduct routine quarterly visits once the newborns are stabilized beyond the neonatal stage. They also stressed the importance of providing PRN nurses for secondary review in cases where complex issues arise beyond the scope of CHPs' training. Additionally, regarding alternative approaches to overcome resource constraints—such as transport costs, a key driver of the RN model—they suggested alternatives like telehealth, similar to the approach used in our research model during the COVID-19 pandemic, to help address this barrier (further elaborated in our qualitative manuscript in preparation on policymakers' perspectives on scaling up postnatal home visits in Kenya).

They further emphasized the need for government and program funders to ensure that they can meet the increased demand for facility health service utilization that would arise from the uptake of this community intervention at scale, following strengthened care at the community level. To maximize the impact of scaling up this community-based intervention, there is a requirement for corresponding improvements in facility-based quality of care (separate manuscript in progress).

In terms of Cost Positioning, Kenya's CHP-only model, with an annual cost of $26.51 per visit, aligns well with international benchmarks for community health worker programs. This is comparable to Ethiopia's model ($27.00), reinforcing the viability of CHP-led delivery. In contrast, Kenya's RN-only model is more expensive at $36.45 per visit, exceeding costs reported in most other settings. Regarding training Intensity and cost, higher training intensity correlates with increased program costs. For example, Malawi's 10–12 weeks of training plus facility experience results in higher annual costs. Kenya's standard CHP training is similar to that in Ghana and Tanzania, demonstrating that adequate preparation can be achieved without high expenditure. Considering Scalability, global examples support the cost-efficiency of the CHP-only model. Bolivia's approach, leveraging existing personnel with minimal new investment, shows affordable scale-up. Conversely, South Africa's high-cost model ($35,714 per worker annually) highlights the financial burden of more intensive systems. Finally, reviewing Intervention Scope Alignment, Kenya's focus on preventive care, with referral linkages, aligns with proven international models. The hybrid model, integrating CHPs and RNs, offers a flexible solution that balances cost and quality, reflecting global best practices. This comparative analysis provides strong support for Kenya's policy direction and is summarized in Supplementary Table S4, which includes cost and programmatic comparisons globally.

Although our study does not include direct measures of health outcomes such as reductions in neonatal mortality, similar community-based postnatal interventions have demonstrated strong potential for cost-effectiveness, providing useful context for interpreting our results. For instance, the COMBINE trial in Ethiopia reported incremental costs per home visit of USD 27 in the control arm and USD 34 in the intervention arm—figures that align closely with our modeled range of USD 26.51 to USD 52.12, depending on provider mix. In COMBINE, the addition of community-level management of possible serious bacterial infections (PSBI) was associated with a 17% reduction in neonatal mortality after day one and resulted in an estimated cost per DALY averted of USD 223, equivalent to approximately 47% of Ethiopia's GDP per capita—meeting WHO-CHOICE crieria for a highly cost-effective intervention.

Our findings also compare favorably with those from Karin Stenberg et al. (2021), who conducted a Generalized Cost-Effectiveness Analysis (GCEA) for a range of newborn health interventions [including home visits for clean postpartum practices, clean cord care (clean birth practices), community-based newborn and child care, Kangaroo mother care, promotion of breastfeeding, and infant and young child feeding] across varying coverage levels. This analysis found that all seven newborn-specific interventions produced average cost-effectiveness ratios (ACERs) below I$100 per healthy life year (HLY) gained. Notably, the home visit intervention for promoting clean postnatal care practices had an ACER of just I$11.5/HLY gained, making it one of the most cost-effective interventions assessed. While our study did not include outcome data necessary for estimating ICERs or HLYs directly, our cost estimates suggest that similar home visit interventions—if achieving modest health gains—could yield comparably favorable cost-effectiveness results.

Furthermore, their analysis of maternal, newborn, and child health interventions in Eastern sub-Saharan Africa and South-East Asia reported ACERs ranging from USD 82 to USD 379 per DALY averted. These estimates were based on a generalized modeling approach assuming no existing intervention comparator, in contrast to ICERs, which compare incremental cost and benefit relative to an existing standard. Even within that framework, our modeled unit costs are well within the range of costs for interventions considered cost-effective under WHO-CHOICE thresholds.

## 5 Strengths and limitations

Our analysis has several strengths and some limitations to note.

**Strengths** include the Use of real-world data: the costing is based on actual expenditures from our longitudinal study, which lends credibility and grounding to our modeling assumptions. The use of real-time budget data from carefully maintained and detailed research study expense records provides a reproducible template for future programs and research projects. Flexible Costing Tool: The inclusion of an Excel-based calculator and generalized equation (Equation 1) provides a valuable resource for policymakers and health planners to adapt the model to different scenarios. Engagement with Policymakers: Early and ongoing engagement with health officials through an iterative process enhances the contextual validity and policy applicability of the findings.

### 5.1 Limitations

Our analysis relies on retrospective cost data, which prevents us from conducting a societal perspective assessment. Such an analysis would have required prospective data collection from families to estimate costs foregone by mothers and families receiving the intervention. Since this was an observational study with very low mortality rates, we were unable to perform a cost-effectiveness analysis. Nevertheless, we collected extensive clinical data on participants; an analysis comparing child outcomes between women who chose clinic visits only and those who also received home visits will be presented separately. Furthermore, because participants selected whether to receive home visits rather than being randomly assigned, home visits demonstrated high acceptability and retention. This self-selection bias could influence the program's costs; for example, if implemented in the community, participants might need more phone calls, tracing, or follow-up visits. Similarly, the eligibility criteria resulted in a cohort of women without complex obstetric or health issues; the broader population is likely to be more diverse, with more women experiencing complicated pregnancies, deliveries, or newborn health issues that could necessitate additional follow-up visits and increase program costs.

While a formal ICER analysis was not feasible in our study due to the lack of outcome data and low mortality rates, our costing framework is well positioned to inform future cost-effectiveness modeling. We believe this addition improves alignment with broader economic evaluation frameworks and enhances the interpretability of our results for decision-makers.

## 6 Conclusions

Our study provides resources to support efforts to increase investment in postnatal home visit programs as policymakers evaluate the costs and benefits of these critical strategic decisions. By estimating the costs of three different staffing approaches, we offer tools for providers to consider various costing scenarios to meet the needs of diverse populations. These findings contribute to ongoing policy discussions on optimizing postnatal home visit programs within resource-limited settings. Future research should examine the cost-effectiveness of home visits in terms of maternal and neonatal health outcomes, as well as strategies for integrating postnatal home visits into existing community health programs in a financially sustainable way. Strengthening government investment in postnatal care—through budget allocation and strategic task-sharing between CHPs and nurses—will be critical for effectively scaling up these interventions and improving newborn and maternal health outcomes.

## Data Availability

The original contributions presented in the study are included in the article/Supplementary Material, further inquiries can be directed to the corresponding authors.
