# Supplementary material for: A cost analysis of postpartum home visit programming in Kenya: estimates to aid policymakers
Source: Front Health Serv. 2025 Nov 13;5:1644078. doi: 10.3389/frhs.2025.1644078 (PMC12657431; doi:10.3389/frhs.2025.1644078)
Supplement: Supplementary file 1 [file Table1.docx]

**SUPPLEMENTARY FILE CHECK LIST:**

|  | **Item** | **Description** | **Yes/No** |
| --- | --- | --- | --- |
| 1 | [Supplemental File 1](https://docs.google.com/spreadsheets/d/1DuCk2xlcgmiBXuvNILbAKyFuPau2D_vf/edit?gid=1317430870#gid=1317430870) | Excel Sheet calculating the cost of postnatal home visits in a research setting | Yes |
| 2 | [Supplemental File 2](https://docs.google.com/spreadsheets/d/1AoqNT_5W9gKMGap-qxfiB7ZBQmy_HdlUjnIypUuhNyY/edit?gid=897414543#gid=897414543) | Excel sheet outlining the calculation of the payer as the Government for postnatal home visits, comparing three staffing models (CHP only, RN only, combined, and Hybrid) with a customizable calculator | Yes |
| 3 | [Supplementary File 3](https://docs.google.com/document/d/1L3pL5J6nH0jKOj36Zod-L2A-zxcYjGQllT4DeGZKi8U/edit?tab=t.0) | Cost categories are outlined assumptions Tables 4a,4b, and 5 | Yes |
| 4 | [Supplementary file 4](https://docs.google.com/document/d/1SXAK76Qo6_P-FOlQHG6JCZMF9ss9X3neRe4TH_5a2ic/edit?tab=t.0) | How Linda Kizazi's cost calculator integrates with existing frameworks, an example of WHO CHOICE | Yes |
| 5 | [Supplementary File 5](https://docs.google.com/document/d/1osxuaDo7Tp-ZSR7USkzOjt0od2aYDrQbt6Gmj-wxXpo/edit?tab=t.0) | CHEERS checklist | Yes |
