# Supplementary material for: A cost analysis of postpartum home visit programming in Kenya: estimates to aid policymakers
Source: Front Health Serv. 2025 Nov 13;5:1644078. doi: 10.3389/frhs.2025.1644078 (PMC12657431; doi:10.3389/frhs.2025.1644078)
Supplement: Supplementary file 4 [file Table4.docx]

**COSTING METHODOLOGY AND ASSUMPTIONS FOR POST-NATAL HOME VISITS IN LINDA KIZAZI STUDY**

**Table 4a. Key Components of Costing Models**

| **Component** | **Details** |
| --- | --- |
| Resources Identified & Categorized | **Capital Resources**: Buildings, vehicles, equipment, and one-off training costs (valued over USD 100).  **Recurrent Costs:** Personnel (salaries and benefits), recurrent training, medical/non-medical supplies, transport (hired/owned), and facility maintenance. Personnel costs were calculated using the Annual Fixed-Term Equivalent (FTE) method. |
| Time Horizon | 12-month period (FY 2019) |
| Payer Perspective | Costs analyzed from research study and government perspectives. |
| Shared Resources | Resources used across multiple cost categories (e.g., facilities, equipment) were allocated proportionately based on the ratio of home visits to total visits. |
| Financial Data | Extracted from the Linda Kizazi Study financial records, including capital assets (buildings, vehicles, equipment). |
| Data Collection | Interviews with study and health facility staff captured time spent on postnatal home visits. |
| Tracked Visits | 1,116 postnatal home visits were recorded for cost calculations. For government modeling, the same number of visits was used across three models: RN, hybrid (RN+CHP), and CHP. |
| Direct Costs | Training, transport, supplies, equipment, personnel, and mobilization. |
| Comparison with Government Costs | Personnel salaries compared to the Kenya Ministry of Health rates. |
| Inflation Adjustments | Costs adjusted for inflation to reflect current values. |
| Government Cost Modeling | Used the ingredients approach, where recurrent costs varied while maintaining capital costs constant. |
| Costing Period & Perspective | Policymakers provided feedback on three models: RN, hybrid (RN+CHP), and CHP to assess feasibility and scalability. |

**Table 4b.Details of components in costing**

| **Component** | **Details and assumptions***(Add this)** |
| --- | --- |
| Personnel | Salaries and benefits for CHPs, nurses, and other health workers |
| Transport | Travel costs for home visits, including fuel and vehicle maintenance |
| Supplies | Medical supplies, protective equipment, and data collection tools |
| Training | Costs associated with training CHPs and other staff |
| Supervision | Expenses related to monitoring and evaluating home visits |
| Overheads | Administrative and operational costs to support service delivery |

**Table 5: Guide to costing methodology and assumptions in the research and government models**

| **Cost categories** | **Assumptions/ costing method** | **Allocation criteria/ notes** |  |
| --- | --- | --- | --- |
| **Capital** | | |  |
| Buildings | The container at Mathare North health center was assumed to have a useful life of 25 years (amortization period), similar to that of a building. | From General Linda Kizazi cost, the building cost has been stepped down to Linda Kizazi home visit using the No. of home visits. as a percentage of total visits (home visit + Clinic visits), as the denominator. |  |
| Vehicles | Annualized replacement value: The vehicle, valued at 30% of the annualized value, was assumed to have been used by MTCV. This is treated as a general project cost. | From General project cost 30% of the vehicle’s replacement value has been allocated to the Linda Kizazi project and again allocated to home visit using the No. of home visit as a percentage of total visits (hone visits +Clinic visits) as the denominator. |  |
| Equipment | The training cost was set at $100 or more; a 3% discount rate was assumed. | The general equipment purchase price has been adjusted for inflation and then stepped down using No. of home visits as a percentage of the total visit (Home visit +Clinic visits) as a denominator, however, the cost for equipment specific to home visit intervention has been allocated directly at 100% to the home visit intervention after price adjustment. |  |
| Training, non-recurrent | Training cost of $100 was determined, assuming a 3% discount rate. | The non-recurrent training costs were entirely allocated to the home visit intervention. |  |
| **Recurrent** | | |  |
| Personnel | Total staff time used; Interviewed health staff to determine activity-specific allocation. | Step-down allocation; using the total staff approach; the staff time spent on home visit intervention was multiplied with the annual salary (FTE) allocated directly to the intervention using No. of home visits as % of the total visits (home visit + Clinic visits) as a denominator. |  |
| Utilities, communication | Electricity, water, sewer, internet, telephone, mail, and insurance (excluding vehicle or staff) costs were determined by month or year depending on availability. | The General project costs of utilities & communication has been stepped down using the No. of home visits as a percentage of the total visits (Home visit + Clinic visits) as denominator. |  |
| Supplies, non-medical | To be costed for each home vs clinic visit | Step-down allocation using the No. of home visits as percentage of total visits (Home visit + Clinic visits) as denominator. |  |
| Maintenance /Repairs | Office building repairs were assumed to be paid together with the rent. Equipment and vehicle repairs/maintenance were assumed to be 10% of their replacement value. | The general Linda Kizazi project cost is stepped down to home visit intervention using No. of home visits as a percentage of the total visits (home visits + Clinic visits) as a denominator. |  |
| Training/meetings, recurrent | No recurrent (additional or follow-up ) trainings were conducted after the initial training. |  |  |
| Transport | Included fuel and vehicle insurance; allocated per annum transport cost to each intervention | Allocated directly to home visit intervention using No. of visits as % of the total visits (home visits + clinic visits) as a denominator |  |
| Home Versus Clinic-Related Costs | The Resource utilization by a Home or Clinic visit is assumed to be proportional to the number of home and clinic visits conducted. | Home & Clinic visit-specific costs were be allocated directly at 100% to the Linda Kizazi Home & Clinic visit. Shared costs between Home & Clinic Visit were allocated to the home visit at 82.7%, based on the number of home visits in 2019 (1116) as a proportion of the total visits for Linda Kizazi Project in 2019 (1350), and at 17.3% to the Clinic visit, based on the number of clinic visits in 2019 (234) as a proportion of all visits for Linda Kizazi Project in 2019 (1350). |  |
|  |  |  |  |
|  |  |  |  |
| Government Models’ Comparison Analysis (Registered Nurse-RN, Community Health Promoter- CHP, Combined) | For Models Costs computations, the assumption will be that the Capital costs are assumed to be fixed (Fixed Cost FC) and Recurrent costs are assumed to be Variable (Variable Cost VC);  Therefore, the Total cost for each model will be calculated as follows: TC = FC + VC. | For all the variable costs (VC), the MOH payment rates are used for:  1. Personnel;  2. Current Recommended Cost (CRC) salary scale for nurses working in level III health facilities;  3. Transport; reimbursement based on CRC rates for a similar cadre of staff;  4. Supplies: cost of supplies used for similar activities, as well as for communication and utilities.  5. Fixed costs, building, vehicles, and other capital items will remain the same as those calculated for the Linda Kizazi Project. |  |
|  |  |  |  |
|  |  |  |  |
