# Supplementary material for: A cost analysis of postpartum home visit programming in Kenya: estimates to aid policymakers
Source: Front Health Serv. 2025 Nov 13;5:1644078. doi: 10.3389/frhs.2025.1644078 (PMC12657431; doi:10.3389/frhs.2025.1644078)
Supplement: Supplementary file 5 [file Table5.docx]

**HOW DOES LINDA KIZAZI'S COST CALCULATOR INTEGRATE WITH EXISTING FRAMEWORKS EXAMPLE WHO CHOICE**

| **Key differences** | **Program scale-up Cost calculator from Linda Kizazi** | **WHO CHOICE Calculator** |
| --- | --- | --- |
| **Level of analysis** | Our study cost calculator is micro-level (single program/single study) | WHO CHOICE is macro-level (health system). |
| **Intended audience** | The cost calculator is designed for researchers and institutions; therefore, our findings are complementary to those of WHO CHOICE, not a substitute. | WHO CHOICE is for policymakers and health system planners |
| **Utility** | Our cost calculator's utility is for financial planning and budgeting | WHO CHOICE is used for priority setting and resource allocation in healthcare. |
| **Characteristics** | **Program scale up cost calculator** | **WHO CHOICE Calculator** |
| **Overall distinction in purpose and audience** | Our research study cost calculator was designed to estimate the costs associated with a specific program for scale-up or intervention, using our example of postnatal home visits for a particular research project. It was then used to develop a customizable tool to help policymakers interested in scaling up postnatal home visits. This tool allows for comparison of various staffing approaches but does not determine cost-effectiveness and is therefore complementary to WHO CHOICE, which caters for cost-effectiveness | WHO CHOICE, on the other hand, is a broader initiative by the World Health Organization focused on helping countries prioritize healthcare interventions based on cost-effectiveness |
| **Focus** | An individual program or project example for research, providing cost estimates for specific interventions in our example postnatal home visits, and is adaptable for different administrative units or counties in Kenya at various levels of the health system, from level 1 to 6 | Healthcare systems and public health interventions. |
| **Purpose** | Our proposed scale-up program cost calculator can be used at the local level, such as an institution like a research project or health facility or within country administrative units like counties, for budgeting, financial planning, and resource allocation for a specific program, for example, postnatal home visits in our study, and is therefore complementary to WHO CHOICE. | Guiding health policy and resource distribution decisions at a national or regional level for example scaling up the post natal home visits program nationwide |
| **Scope** | Narrow, focusing on the costs of a single program or intervention, in this case, postnatal home visits; limited in the calculation of cost effectiveness, and therefore complementary to WHO CHOICE, which determines cost effectiveness | Broadly focusing on examining the cost-effectiveness of various healthcare interventions across different settings. |
| **Example** | A new program for scale-up planning or a research team planning a clinical trial can utilize a cost calculator to estimate expenses, such as participant recruitment, drug costs, and data analysis. | A government can use WHO CHOICE to decide whether to invest in preventative programs like vaccination campaigns or treatment programs for specific diseases. |
