# Supplementary material for: A cost analysis of postpartum home visit programming in Kenya: estimates to aid policymakers
Source: Front Health Serv. 2025 Nov 13;5:1644078. doi: 10.3389/frhs.2025.1644078 (PMC12657431; doi:10.3389/frhs.2025.1644078)
Supplement: Supplementary file 8 [file Datasheet1.pdf]

SUPPLEMENTARY FILE CHECK LIST:

	**Item**	**Description**	**Yes/No**
1	Supplemental File 1	Excel Sheet calculating the cost of postnatal home visits in a research setting	Yes
2	Supplemental File 2	Excel sheet outlining the calculation of the payer as the Government for postnatal home visits, comparing three staffing models (CHP only, RN only, combined, and Hybrid) with a customizable calculator	Yes
3	Supplementary File 3	Cost categories are outlined assumptions Tables 4a,4b, and 5	Yes
4	Supplementary file 4	How Linda Kizazi's cost calculator integrates with existing frameworks, an example of WHO CHOICE	Yes
5	Supplementary File 5	CHEERS checklist	Yes
