# Supplementary material for: A cost analysis of postpartum home visit programming in Kenya: estimates to aid policymakers
Source: Front Health Serv. 2025 Nov 13;5:1644078. doi: 10.3389/frhs.2025.1644078 (PMC12657431; doi:10.3389/frhs.2025.1644078)
Supplement: Supplementary file 9 [file Datasheet2.pdf]

COSTING METHODOLOGY AND ASSUMPTIONS FOR POST-NATAL HOME VISITS IN LINDA KIZAZI STUDY

Table 4a. Key Components of Costing Models

Component	Details
Resources Identified & Categorized	**Capital Resources**: Buildings, vehicles, equipment, and one-off training costs (valued over USD 100). **Recurrent Costs:** Personnel (salaries and benefits), recurrent training, medical/non-medical supplies, transport (hired/owned), and facility maintenance. Personnel costs were calculated using the Annual Fixed-Term Equivalent (FTE) method.
Time Horizon	12-month period (FY 2019)
Payer Perspective	Costs analyzed from research study and government perspectives.
Shared Resources	Resources used across multiple cost categories (e.g., facilities, equipment) were allocated proportionately based on the ratio of home visits to total visits.
Financial Data	Extracted from the Linda Kizazi Study financial records, including capital assets (buildings, vehicles, equipment).
Data Collection	Interviews with study and health facility staff captured time spent on postnatal home visits.
Tracked Visits	1,116 postnatal home visits were recorded for cost calculations. For government modeling, the same number of visits was used across three models: RN, hybrid (RN+CHP), and CHP.
Direct Costs	Training, transport, supplies, equipment, personnel, and mobilization.
Comparison with Government Costs	Personnel salaries compared to the Kenya Ministry of Health rates.
Inflation Adjustments	Costs adjusted for inflation to reflect current values.
Government Cost Modeling	Used the ingredients approach, where recurrent costs varied while maintaining capital costs constant.
Costing Period & Perspective	Policymakers provided feedback on three models: RN, hybrid (RN+CHP), and CHP to assess feasibility and scalability.

Table 4b.Details of components in costing

Component	Details and assumptions***(Add this)
Personnel	Salaries and benefits for CHPs, nurses, and other health workers
Transport	Travel costs for home visits, including fuel and vehicle maintenance
Supplies	Medical supplies, protective equipment, and data collection tools
Training	Costs associated with training CHPs and other staff
Supervision	Expenses related to monitoring and evaluating home visits
Overheads	Administrative and operational costs to support service delivery

Table 5: Guide to costing methodology and assumptions in the research and government models

Cost categories	Assumptions/ costing method	Allocation criteria/ notes
**Capital**		
Buildings	The container at Mathare North health center was assumed to have a useful life of 25 years (amortization period), similar to that of a building.	From General Linda Kizazi cost, the building cost has been stepped down to Linda Kizazi home visit using the No. of home visits. as a percentage of total visits (home visit + Clinic visits), as the denominator.
Vehicles	Annualized replacement value: The vehicle, valued at 30% of the annualized value, was assumed to have been used by MTCV. This is treated as a general project cost.	From General project cost 30% of the vehicle's replacement value has been allocated to the Linda Kizazi project and again allocated to home visit using the No. of home visit as a percentage of total visits (hone visits +Clinic visits) as the denominator.
Equipment	The training cost was set at $100 or more; a 3% discount rate was assumed.	The general equipment purchase price has been adjusted for inflation and then stepped down using No. of home visits as a percentage of the total visit (Home visit +Clinic visits) as a denominator, however, the cost for equipment specific to home visit intervention

		has been allocated directly at 100% to the home visit intervention after price adjustment.
Training, non-recurrent	Training cost of $100 was determined, assuming a 3% discount rate.	The non-recurrent training costs were entirely allocated to the home visit intervention.
**Recurrent**		
Personnel	Total staff time used; Interviewed health staff to determine activity-specific allocation.	Step-down allocation; using the total staff approach; the staff time spent on home visit intervention was multiplied with the annual salary (FTE) allocated directly to the intervention using No. of home visits as % of the total visits (home visit + Clinic visits) as a denominator.
Utilities, communication	Electricity, water, sewer, internet, telephone, mail, and insurance (excluding vehicle or staff) costs were determined by month or year depending on availability.	The General project costs of utilities & communication has been stepped down using the No. of home visits as a percentage of the total visits (Home visit + Clinic visits) as denominator.
Supplies, non-medical	To be costed for each home vs clinic visit	Step-down allocation using the No. of home visits as percentage of total visits (Home visit + Clinic visits) as denominator.
Maintenance /Repairs	Office building repairs were assumed to be paid together with the rent. Equipment and vehicle repairs/maintenance were assumed to be 10% of their replacement value.	The general Linda Kizazi project cost is stepped down to home visit intervention using No. of home visits as a percentage of the total visits (home visits + Clinic visits) as a denominator.
Training/meetings, recurrent	No recurrent (additional or follow-up) trainings were conducted after the initial training.	
Transport	Included fuel and vehicle insurance; allocated per annum transport cost to each intervention	Allocated directly to home visit intervention using No. of visits as % of the total visits (home visits + clinic visits) as a denominator

Home Versus Clinic-Related Costs	The Resource utilization by a Home or Clinic visit is assumed to be proportional to the number of home and clinic visits conducted.	Home & Clinic visit-specific costs were be allocated directly at 100% to the Linda Kizazi Home & Clinic visit. Shared costs between Home & Clinic Visit were allocated to the home visit at 82.7%, based on the number of home visits in 2019 (1116) as a proportion of the total visits for Linda Kizazi Project in 2019 (1350), and at 17.3% to the Clinic visit, based on the number of clinic visits in 2019 (234) as a proportion of all visits for Linda Kizazi Project in 2019 (1350).
Government Models' Comparison Analysis (Registered Nurse-RN, Community Health Promoter- CHP, Combined)	For Models Costs computations, the assumption will be that the Capital costs are assumed to be fixed (Fixed Cost FC) and Recurrent costs are assumed to be Variable (Variable Cost VC);<br>Therefore, the Total cost for each model will be calculated as follows: TC = FC + VC.	For all the variable costs (VC), the MOH payment rates are used for:<br>1. Personnel;<br>2. Current Recommended Cost (CRC) salary scale for nurses working in level III health facilities;<br>3. Transport; reimbursement based on CRC rates for a similar cadre of staff;<br>4. Supplies: cost of supplies used for similar activities, as well as for communication and utilities.<br>5. Fixed costs, building, vehicles, and other capital items will remain the same as those calculated for the Linda Kizazi Project.
